# Mitigation of Paddy Field Soil Methane Emissions by Betaproteobacterium *Azoarcus* Inoculation of Rice Seeds

**DOI:** 10.1264/jsme2.ME22052

**Published:** 2022-12-14

**Authors:** Midori Sakoda, Takeshi Tokida, Yoriko Sakai, Keishi Senoo, Tomoyasu Nishizawa

**Affiliations:** 1 United Graduate School of Agricultural Science, Tokyo University of Agriculture and Technology, Tokyo 183–8509, Japan; 2 Institute for Agro-Environmental Sciences, National Agriculture and Food Research Organization, Ibaraki 305–8604, Japan; 3 Graduate School of Agricultural and Life Sciences, The University of Tokyo, Tokyo 113–8657, Japan; 4 Collaborative Research Institute for Innovative Microbiology, The University of Tokyo, Tokyo 113–8657, Japan; 5 Ibaraki University College of Agriculture, Ibaraki 300–0393, Japan

**Keywords:** methane, paddy field, *Azoarcus*, greenhouse gas, mitigation technique

## Abstract

Paddy fields are a major source of atmospheric methane, a greenhouse gas produced by methanogens and consumed by methanotrophs in flooded soil. The inoculation of rice seeds with the bacterium *Azoarcus* sp. KH32C alters the rice root-associated soil bacterial community composition. The present study investigated the effects of KH32C-inoculated rice cultivation on soil methanogens and methanotrophs involved in methane emissions from a rice paddy field. KH32C-inoculated and non-inoculated rice (cv. Nipponbare) were cultivated in a Japanese rice paddy with and without nitrogen fertilizer. Measurements of methane emissions and soil solution chemical properties revealed increases in methane flux over the waterlogged period with elevations in the concentrations of dissolved methane, dissolved organic carbon, and ferrous iron, which is an indicator of soil reduction levels. Reverse transcription quantitative PCR and amplicon sequencing were used to assess the transcription of the methyl-coenzyme M reductase gene (*mcrA*) from methanogens and the particulate methane monooxygenase gene (*pmoA*) from methanotrophs in paddy soil. The results obtained showed not only the transcript copy numbers, but also the compositions of *mcrA* and *pmoA* transcripts were related to methane flux. KH32C-inoculated rice cultivation recruited soil methanogens and methanotrophs that suppressed high methane synthesis, increased methane consumption, and decreased methane emissions by 23.5 and 17.2% under non-fertilized and nitrogen-fertilized conditions, respectively, while maintaining rice grain yield. The present study demonstrated the mitigation of paddy field methane emissions arising from the use of KH32C in rice cultivation due to its influence on the compositions of soil methanogen and methanotroph populations.

The global warming potential of methane, a major greenhouse gas, is 34-fold higher than that of carbon dioxide (CO_2_) (Myhre *et al.*, 2013). In 2019, global atmospheric methane concentrations reached 1,877±2 ppb, which is 260% of pre-industrial levels (World Meteorological Organization, 2020). Rice paddy fields are a major source of methane emissions, and are estimated to account for 8% of total global anthropogenic methane emissions (Saunois *et al.*, 2020). In Asia, where rice is a staple food, rice cultivation methane emissions in 2017 accounted for 22.4 Tg, which is approximately 35% of total agricultural methane emissions (FAOSTAT 2020, http://www.fao.org/faostat/en/#home). In Japan, methane emissions from rice cultivation account for 42% of total methane emissions (Greenhouse Gas Inventory Office of Japan and Ministry of the Environment, Japan, 2021). Therefore, reductions in paddy field methane emissions are essential to mitigate climate change.

Paddy field methane emissions are affected by several factors, including fertilization (Kesheng and Zhen, 1997; Xie *et al.*, 2010; Dong *et al.*, 2011; Banger *et al.*, 2012; Yuan *et al.*, 2018), cultivars (Watanabe *et al.*, 1995; Kesheng and Zhen, 1997; Wang *et al.*, 1997), and atmospheric CO_2_ concentrations (Inubushi *et al.*, 2003). The treatment of paddy fields with iron effectively mitigates methane emissions (Jäckel *et al.*, 2005; Liu *et al.*, 2012; Susilawati *et al.*, 2015); however, this is associated with a risk of iron toxicity, which may inhibit rice growth (Jäckel and Schnell, 2000). Water management also affects paddy field methane emissions; prolonged midseason drainage has been shown to effectively mitigate methane emissions (Itoh *et al.*, 2011). Methane emissions are produced and oxidized by the soil microbial community, which is affected by the rice cultivation regimen and growth stage. The copy numbers and compositions of the genes and transcripts involved in microbial methane production and oxidization have been implicated in methane emissions (Ma *et al.*, 2010; Lee *et al.*, 2014; Liu *et al.*, 2016; Yuan *et al.*, 2018). In flooded soil, methanogenesis is the final step in the anaerobic microbial degradation of organic matter. Microbes degrade soil organic matter and fresh organic matter, *e.g.*, rice straw and rhizodeposition, into acetate, CO_2_, and hydrogen, and methanogens then produce methane using acetate or CO_2_ and hydrogen (Conrad, 2020). Conversely, methanotrophs oxidize methane (Hanson and Hanson, 1996; Le Mer and Roger, 2001). Methane emitted from paddy fields is mostly released from the rhizosphere into the atmosphere through rice (Cicerone and Jolla, 1981; Holzapfel-Pschorn *et al.*, 1986). Methanotrophs appear to be an effective mitigation method for methane emissions. Davamani *et al.* (2020) reported that the spreading of carrier-based methanotrophs along with nitrogen-fixing bacteria and phosphate-solubilizing bacteria onto paddy fields prior to rice transplantation in India mitigated methane emissions. Furthermore, Rani *et al.* (2021) showed that several inoculations of rice with methanotrophic bacteria, before and after transplantation, reduced methane emissions from paddy fields when chemical and organic fertilizers were applied.

The betaproteobacterium* Azoarcus* exerts positive effects on plant growth (Hurek *et al.*, 2002; Fernández *et al.*, 2014) and degrades aromatic compounds (Anders *et al.*, 1995; López Barragán *et al.*, 2004; Rabus *et al.*, 2005). The complete genome sequencing of *Azoarcus* sp. KH32C, obtained from a paddy upland rotation field in Japan (Tago *et al.*, 2011), revealed the absence of indole-pyruvic acid biosynthesis genes, which are plant growth-promoting factors. However, the genome sequence showed that KH32C possesses genes that encode for capsular polysaccharide biosynthesis, which is associated with plant-microbe interactions (Nishizawa *et al.*, 2012). We previously demonstrated that rice seed inoculation with KH32C, which colonized rice seedling roots, improved the early growth phase growth rate and appeared to increase the concentration of zinc in brown rice without affecting grain yield in low nitrogen input paddy fields (Sakoda *et al.*, 2019). Furthermore, a bacterial 16S rRNA gene-based amplicon sequencing ana­lysis revealed that a rice seed inoculation with KH32C affected the rice root-associated soil bacterial community, including potential plant growth-promoting bacteria. In this amplicon sequencing ana­lysis, only one amplicon sequence (0.003%) of the genus *Azoarcus* was detected in all amplicon sequences of the harvesting stage (Sakoda *et al.*, 2019); the observed effect was similar to “priming”. “Priming” demonstrated that the application of beneficial bacteria may stimulate plants and alter responses to biotic and abiotic stresses (Conrath *et al.*, 2006; Bruce *et al.*, 2007). Since soil microbes are key components in the biogeochemical cycle, a population shift in these microbes may influence this cycle; however, the effects of inoculating rice seeds with beneficial bacteria remain unknown. Particularly, the microbial population changes induced in the biogeochemical cycle of paddy soil included methane production and consumption. The elucidation of these effects is key for the eco-friendly use of beneficial bacteria in agriculture.

Therefore, the present study investigated the effects of cultivating rice inoculated with KH32C on the soil microbial community involved in paddy field methane emissions. We hypothesized that the KH32C inoculation of rice seeds may affect rice paddy soil methane-producing and -oxidizing microbes, thereby mitigating methane emissions. To verify this, we examined the transcription of genes related to methanogenesis and methane oxidation in rice root-associated soil and measured methane emissions from fertilized and non-fertilized paddy field areas in which rice inoculated with KH32C was cultivated.

## Materials and Methods

### Bacterium culture and bacterium inoculation onto rice seeds

*Azoarcus* sp. KH32C was cultured on a DNB-NS agar plate (0.08‍ ‍g nutrient broth, 3.0‍ ‍mM sodium nitrate, 4.4‍ ‍mM sodium succinate, and 15‍ ‍g agar L^–1^; Tago *et al.*, 2011) at 27°C under anaerobic conditions (Sakoda *et al.*, 2019).

*Oryza sativa* L. cv. Nipponbare was used in the present study. Pregerminated rice seeds were immersed in a KH32C cell suspension diluted with sterilized water containing 50‍ ‍μM calcium chloride (CaCl_2_), in which the cell concentration was adjusted to approximately 1.3×10^5^‍ ‍cells‍ ‍mL^–1^, for 10‍ ‍min (Sakoda *et al.*, 2019). In a control experiment with a non-bacterium inoculation, pregerminated rice seeds were immersed in sterilized water containing 50‍ ‍μM CaCl_2_.

### Rice cultivation and investigation of grain yield

Rice cultivation was examined at the Institute for Agro-Environmental Sciences, National Agriculture and Food Research Organization (36°01′N, 140°07′E) in 2019. Pregerminated rice seeds with and without KH32C were sown in seedling trays on May 7^th^. Seedlings were transplanted to a paddy field (Human-Made soils, pH 5.8) on 29^th^ May. The planting density was 22.2‍ ‍hills‍ ‍m^–2^ with three seedlings per hill. Our experimental site was a split-plot design in triplicate. Two levels of nitrogen fertilizer were used as the main factor: 0‍ ‍g N m^–2^ (0N) and 8‍ ‍g N m^–2^ (8N). In the 8N plots, nitrogen fertilizer was applied as a basal dressing at a rate of 2‍ ‍g N m^–2^ as urea (Mitsui Chemicals) and coated urea, LP40, LP100, and LP140 (JCAM AGRI.) at 2‍ ‍g N m^–2^ each. Cultivation plots with KH32C-inoculated rice (KH) and non-inoculated rice (CT) were assigned as split factors (Fig. S1a). The paddy field was flooded continuously from before transplantation to the last sampling of gas and soil solutions on September 6^th^.

Rice shoots were sampled on June 25^th^ (27 days after transplantation [DAT]; tillering stage), July 22^nd^ (54 DAT; panicle formation stage), August 14^th^ (77 DAT; heading stage), and August 26^th^ (89 DAT; mid-ripening stage). Six hills were sampled from each plot on each sampling day (Fig. S1b). Sampled rice shoots were dried at 80°C and dry weight was measured.

Rice grains were harvested on September 20^th^ (114 DAT). The grains from nine hills in each plot were sampled for grain yield investigations. After air-drying, husked rice was hulled. Brown rice was dried at 105°C and then weighed. Brown rice yield was calculated using dry weight data and assuming a moisture content of 14%.

### Soil solution, gas, and soil sampling

Soil solutions were sampled using a microporous tube (Cheng *et al.*, 2006) on June 27^th^ (29 DAT; tillering stage), July 10^th^ (42 DAT), July 25^th^ (57 DAT; panicle initiation stage), August 9^th^ (72 DAT; booting stage), August 22^nd^ (85 DAT; mid-ripening stage), and September 6^th^ (100 DAT) 2019. A microporous tube, 10‍ ‍cm in length, was placed vertically into the soil near a hill. The soil solution was transferred to a semi-vacuum bottle (filled with pure N_2_ gas) through a PVC tube, collecting approximately 6.5‍ ‍mL of the soil solution in several h. In each plot, quadruplicate soil solution samples were collected from four sites adjacent to the hills.

Gas sampling to evaluate paddy field methane emissions was conducted using a closed chamber method on the same days as soil solution sampling. In this gas sampling, a transparent closed-top acrylic chamber was used (Inubushi *et al.*, 2003). The basal area of the chamber was 30×60‍ ‍cm, and height was adjusted to rice height on each sampling day. The gas sampling chamber covered the same hills used for soil solution sampling. Gas sampling into vacuum vial bottles was conducted four times for approximately 30‍ ‍min.

Soil samples were collected using an open-end 50-mL syringe from a depth of 1–10‍ ‍cm in three positions at different distances from the hill (Fig. S1b) on rice shoot sampling days (27, 54, 77, and 89 DAT). On the transplantation day (0 DAT; May 29^th^), soil samples were randomly collected from three positions in each 0N and 8N plot using an open-end 50-mL syringe from a depth of 1–10‍ ‍cm. Soil samples from three positions in each plot were pooled and mixed. Some of the soil samples were frozen immediately using liquid nitrogen and stored at –80°C until used for RNA extraction. Soil samples to examine soil water contents were stored at 4°C until used.

### Soil solution ana­lysis

After soil solution sampling, 1‍ ‍mL of 0.5 M sulfuric acid was immediately added to the soil solution sample (approximately 6.5‍ ‍mL). The methane concentration in the headspace of the soil solution sample was analyzed using a gas chromatograph (GC-14A; Shimadzu) fitted with a flame-ionization detector. The dissolved methane concentration of the soil solution was calculated using the headspace methane concentration (Cheng *et al.*, 2005). The concentration of dissolved organic carbon (DOC) in the soil solution was analyzed using a total organic carbon analyzer (TOC-LCSH; Shimadzu). The ferrous iron (Fe [II]) concentration in the soil solution was analyzed using inductively coupled plasma optical emission spectrometry (720-ES; Agilent Technologies) in accordance with Suda *et al.* (2016). The ana­lysis of dissolved methane and Fe (II) concentrations was conducted for each soil solution sample. The ana­lysis of DOC concentrations was performed using samples collected at 29, 57, 72, and 85 DAT. Concentrations were calculated as the means of four samples from each plot.

### Methane emission ana­lysis

Methane concentrations in gas samples were analyzed using a gas chromatograph (GC-14B; Shimadzu) fitted with a flame-ionization detector. Cumulative methane emissions were calculated using methane flux data. Methane flux on May 29^th^ 2019 (0 DAT) was assumed to be 0‍ ‍mg C m^–2^ h^–1^.

### RNA extraction from soil samples and cDNA library synthesis

RNA was extracted from 2‍ ‍g of soil samples stored at –80°C using an RNeasy PowerSoil Total RNA Kit (QIAGEN) in accordance with the manufacturer’s protocol, and 50‍ ‍μL of resuspended RNA in RNase/DNase-free water was obtained. Extracted RNA was treated with DNase using a TURBO DNA-*free*^TM^ kit (Thermo Fisher Scientific) in accordance with the manufacturer’s protocol and 60‍ ‍μL of DNA-free soil RNA was obtained. cDNA synthesis from DNA-free soil RNA was conducted using PrimeScript^TM^ Reverse Transcriptase (Takara Bio) with random primers (hexadeoxyribonucleotide mixture; Takara Bio) in accordance with the manufacturer’s protocol, except for the addition of 1‍ ‍mM dithiothreitol. DNA-free soil RNA was used at a 1/10 volume of the mixture.

### Reverse transcription quantitative PCR assay of *mcrA* and *pmoA* in soil

Quantitative PCR assays for *mcrA*, which encodes the methyl-coenzyme M reductase alpha subunit possessed by methanogens, and *pmoA*, which encodes the particulate methane monooxygenase beta subunit possessed by methanotrophic bacteria, were conducted using the synthesized cDNA library (StepOnePlus^TM^ Real-Time PCR System; Thermo Fisher Scientific). ME3MF_I (5′-TGTCIGGIGGIGTMGGITTYAC-3′; modified from Nunoura *et al.* [2008]; Sakai *et al.*, unpublished) and ME2mod (5′-TCATBGCRTAGTTNGGRTAGT-3′; Mori *et al.*, 2012) primers were used for PCR of *mcrA*, and A189 (5′-GGNGACTGGGACTTCTGG-3′; Holmes *et al.*, 1995) and mb661 (5′-CCGGMGCAACGTCYTTACC-3′; Costello and Lidstrom, 1999) primers for PCR of *pmoA*. The total volume of each PCR mixture was 20‍ ‍μL, which contained 2.0‍ ‍μL of template cDNA, 20 pmol (for *mcrA*) or 1 pmol (for *pmoA*) of forward and reverse primers, 0.1‍ ‍μL of *TaKaRa Ex Taq*^®^ polymerase Hot Start Version (Takara Bio), 2.0‍ ‍μL of 10×*Ex Taq* Buffer (Mg^2+^ plus), 1.6‍ ‍μL of the dNTP mixture (2.5‍ ‍mM each), 0.4‍ ‍μL of 20‍ ‍mg mL^–1^ bovine serum albumin solution, 0.2‍ ‍μL of ROX Reference Dye (Thermo Fisher Scientific), and 0.3‍ ‍μL of 1,000-fold diluted SYBR Green I Nucleic Acid Stain (Lonza). PCR for *mcrA* was performed under the following conditions: initially 94°C (3‍ ‍min), then 40 cycles of 94°C (15 s), 49°C (30‍ ‍s), 72°C (30‍ ‍s), and 80°C (10 s). PCR of *pmoA* was conducted under the following conditions: initially 94°C (3‍ ‍min), then 40 cycles of 94°C (15 s), 55°C (30‍ ‍s), 72°C (30‍ ‍s), and 80°C (10 s). A melting curve ana­lysis was performed after each assay: 94°C (15 s), 60°C (1‍ ‍min), stepwise denaturation from 60°C to 95°C with 0.3°C s^–1^ increases, and 95°C (15 s). A linearized plasmid with the *Methanosarcina acetivorans* C2A PCR fragment generated with the ME3MF_I/ME2mod primer set and a linearized plasmid for soil *pmoA* clones generated with the A189/mb661 primer set were used as standards. Quantitative PCR assays were repeated twice for both *mcrA* and *pmoA*, and the mean of the two repeats was calculated as the copy number in soil cDNA. To calculate copy numbers per gram dry weight soil, soil samples stored at 4°C were dried at 105°C for 12‍ ‍h and soil water contents were calculated.

### Amplicon sequencing ana­lysis of *mcrA* and *pmoA* in soil cDNA

Amplicon sequencing ana­lyses of *mcrA* and *pmoA* were conducted using the cDNA library synthesized from soil RNA. Two-step tailed PCR was performed to prepare the library for the amplicon sequencing ana­lysis. ME3MF (5′-ATGTCNGGTGGHGTMGGSTTYAC-3′; Nunoura *et al.*, 2008) and ME2r’ (5′-TCATBGCRTAGTTDGGRTAGT-3′; Nunoura *et al.*, 2008) primers for *mcrA*, and A189 and mb661 primers for *pmoA* were used for first PCR, in which primers were attached using overhang adapter sequences. The total volume of each PCR mixture was 50‍ ‍μL, which contained 5.0‍ ‍μL of template cDNA, 50 pmol (for *mcrA*) or 5 pmol (for *pmoA*) of forward and reverse primers, 0.5‍ ‍μL (for *mcrA*) or 0.25‍ ‍μL (for *pmoA*) of *TaKaRa Ex Taq*^®^ polymerase Hot Start Version (Takara Bio), 5.0‍ ‍μL of 10×*Ex Taq* Buffer (Mg^2+^ plus), 4.0‍ ‍μL of the dNTP mixture (2.5‍ ‍mM each), and 0.25‍ ‍μL (for *mcrA*) or 1.0‍ ‍μL (for *pmoA*) of 20‍ ‍mg mL^–1^ bovine serum albumin solution. PCR for *mcrA* was conducted under the following conditions: initially 94°C (3‍ ‍min), then optimal cycles of 94°C (30‍ ‍s), 49°C (30‍ ‍s), and 72°C (40 s), and final extension at 72°C (5‍ ‍min). PCR of *pmoA* was performed under the following conditions: initially 94°C (3‍ ‍min), then optimal cycles of 94°C (30‍ ‍s), 55°C (30‍ ‍s), and 72°C (40 s), and final extension at 72°C (5‍ ‍min). First PCR products were purified using a QIAquick gel extraction kit (QIAGEN) in accordance with the manufacturer’s protocol. Purified PCR amplicons were attached to index sequences using second PCR, and second PCR products were purified using a QIAquick gel extraction kit. Paired-end sequencing (2×300 bp) was conducted using a Miseq system with Miseq Reagent Kit v3 (Illumina).

A sequence data ana­lysis was performed as shown in Fig. S2. The quality control of raw reads was conducted using sickle version 1.33 (Joshi and Fass, 2011; available at https://github.com/najoshi/sickle). Quality-controlled paired-end reads were merged and primer sequences were trimmed using mothur v1.46.1 (Schloss *et al.*, 2009). Low-quality sequences were filtered using mothur and chimeras were removed using the mothur-integrated VSEARCH algorithm (Rognes *et al.*, 2016). The detection and correction of frameshift errors in sequences were performed using FrameBot (Wang *et al.*, 2013), and sequences including stop codons were removed. Sequences were aligned against the *mcrA* and *pmoA* sequences retrieved from the fungene database (Fish *et al.*, 2013) using mothur, and the *mcrA* sequences of the recently proposed *Ca.* Syntrophoarchaeum (Laso-Pérez *et al.*, 2016) were then added to the *mcrA* dataset. Aligned sequences were improved using mothur. Aligned, high-quality sequences were classified into operational taxonomic units (OTUs) with nucleotide cut-off values of 84% for *mcrA* (Yang *et al.*, 2014) and 86% for *pmoA* (Wen *et al.*, 2016). The representative amino acid sequences of major OTUs (>5% mean relative abundance for a triplicate measurement of at least one plot measured on one sampling day) and sequences identified from the NCBI database were used for phylogenetic tree building. Multiple sequence alignment and alignment curation were conducted using MAFFT (Katoh and Standley, 2013) and‍ ‍BMGE (Criscuolo and Gribaldo, 2010), integrated with NGPhylogeny.fr (Lemoine *et al.*, 2019), respectively. A phylogenetic tree was constructed in accordance with the maximum likelihood method using PhyML 3.0 (Guindon *et al.*, 2010) with the selection of a best-fit evolutionary model in accordance with the Akaike Information Criterion using Smart Model Selection (Lefort *et al.*, 2017). The SH-like approximate likelihood-ratio test (aLRT) (Anisimova and Gascuel, 2006) was performed to estimate branch support. MEGA11 (Tamura *et al.*, 2021) was used to render and edit the constructed tree.

### Statistical ana­lysis

A mixed linear model was used to assess the effects of nitrogen fertilization and the KH32C inoculation of rice seeds, and computations were performed using the MIXED Procedure of SAS^®^ studio 3.8 (SAS Institute). Data from 0 DAT were not statistically analyzed.

A heatmap ana­lysis of the relative abundance of the major OTUs of *mcrA* and *pmoA* based on the *Z*-score at each of the four growth stages was conducted using the gplots package (Gregory *et al.*, 2020) in R version 4.1.1 (R Core Team, 2021). Data on the mean of triplicate samples from 27, 54, 77, and 89 DAT were used in this ana­lysis.

Spearman’s rank correlation coefficient between methane flux and the relative abundance of major OTUs of *mcrA* and *pmoA* was calculated using the psych package (Revelle, 2021) in R. Spearman’s rank correlation coefficient among methane flux, shoot biomass, the chemical properties of the soil solution, and the copy numbers of *mcrA* and *pmoA* was also calculated. Data from the four growth stages, namely, the tillering stage (27 or 29 DAT), panicle initiation stage (54 or 57 DAT), booting (72 DAT) or heading stage (77 DAT), and mid-ripening stage (85 or 89 DAT), were pooled (*n*=48) in these ana­lyses.

### Accession number

Raw sequence data obtained in the amplicon sequencing ana­lysis were submitted to DDBJ under accession number DRA013646.

## Results

### Concentrations of dissolved methane, DOC, and Fe (II) in the soil solution

**Fig. 1.**
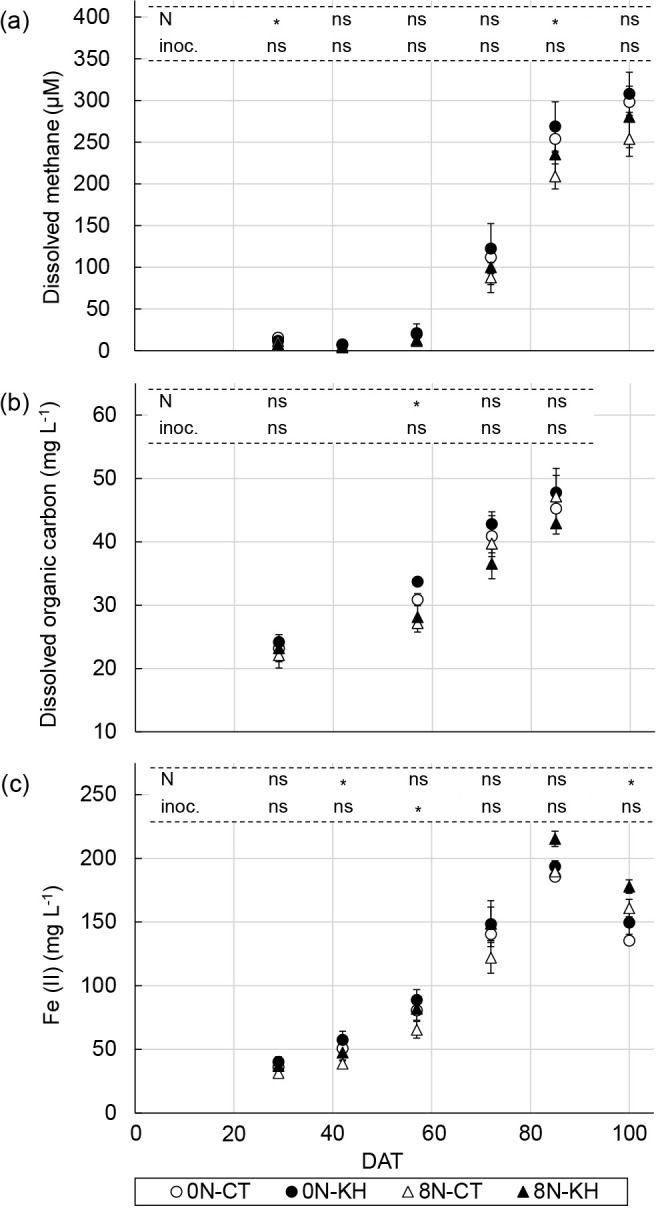
Soil solution chemical properties during rice cultivation. Dissolved methane concentration (a), dissolved organic carbon concentration (b), and ferrous iron concentration (c). Bars represent standard errors (*n*=3). Asterisks above columns indicate a significant difference for each factor tested using a mixed linear model (*P*<0.05). ns indicates no significant difference. Nitrogen fertilization regimen×the KH32C inoculation interaction was not significant for any of the analyzed properties of the soil solutions. 0N, 0‍ ‍g N m^–2^; 8N, 8‍ ‍g N m^–2^; CT, no inoculation; KH, KH32C inoculation; N, nitrogen fertilization regimen; inoc., KH32C inoculation.

Dissolved methane concentrations in the soil solution increased after 72 DAT and continued to increase until 100 DAT across the different treatments. Dissolved methane concentrations were slightly higher in the 0N plots than in the 8N plots, and dissolved methane concentrations at 29 DAT and 85 DAT were significantly lower in the 8N plots than in the 0N plots (*P*<0.05). The effects of the KH32C inoculation were not significant on any sampling day (Fig. 1a).

DOC concentrations in the soil solution gradually increased from 29 DAT to 85 DAT across the different treatments. DOC concentrations at 57 DAT were significantly lower in the 8N plots than in the 0N plots (*P*<0.05). The effects of the KH32C inoculation were not significant on any sampling day (Fig. 1b).

Fe (II) concentrations, an indicator of soil reduction levels, in the soil solution gradually increased from 29 DAT to 85 DAT across the different treatments. Fe (II) concentrations peaked at 85 DAT, and were lower at 100 DAT than at 85 DAT. Fe (II) concentrations at 42 DAT and 100 DAT were significantly lower and higher, respectively, in the 8N plots than in the 0N plots, whereas Fe (II) concentrations at 57 DAT were significantly higher in the KH plots than in the CT plots (*P*<0.05) (Fig. 1c).

The nitrogen fertilization regimen×KH32C inoculation interaction was not significant for any of the analyzed properties of the soil solution samples.

### Methane emissions and rice grain yield

**Fig. 2.**
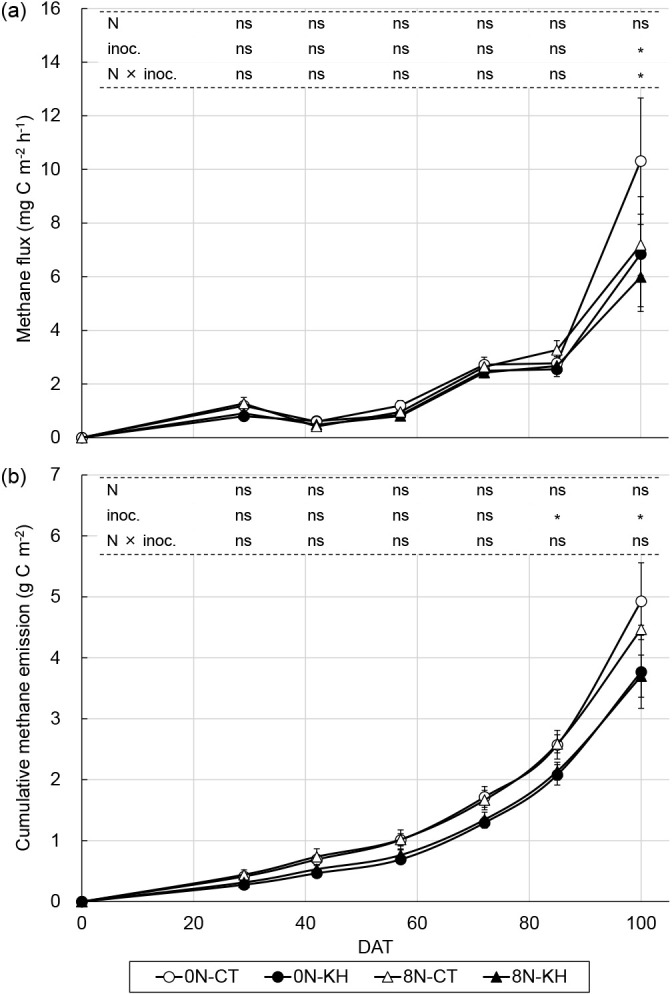
Methane flux (a) and cumulative methane emissions (b) from a paddy field. Bars represent standard errors. Asterisks above the columns indicate a significant difference for each factor tested using a mixed linear model (*P*<0.05). ns indicates no significant difference. 0N, 0‍ ‍g N m^–2^; 8N, 8‍ ‍g N m^–2^; CT, non-inoculation; KH, KH32C inoculation; N, nitrogen fertilization regimen; inoc., KH32C inoculation.

Methane flux from each plot increased after 72 DAT, and increased further at 100 DAT, particularly for the 0N-CT plots (Fig. 2a). Methane flux was 32.9, 29.0, 8.1, 8.2, and 33.6% lower at 29, 57, 72, 85, and 100 DAT, respectively, and 3.2% higher at 42 DAT in the 0N-KH plots than in the 0N-CT plots. Methane flux was 28.8, 15.9, 8.2, 18.2, and 16.7% lower at 29, 57, 72, 85, and 100 DAT, respectively, and 18.0% higher at 42 DAT in the 8N-KH plots than in the 8N-CT plots. Methane flux was significantly lower in the KH plots than in the CT plots at 100 DAT (*P*<0.05). The nitrogen fertilization regimen did not affect methane flux, except at 100 DAT, at which the nitrogen fertilization regimen×KH32C inoculation interaction significantly affected methane flux (*P*<0.05). No significant differences were observed on other sampling days, except 100 DAT (Fig. 2a). The cumulative methane emissions of the KH plots were lower than those of the CT plots for both the 0N and 8N plots. At 85 DAT, cumulative KH plot methane emissions were significantly lower than those of CT plots by 19.1% for the 0N plots and 17.3% for the 8N plots (*P*<0.05). Total methane emissions (cumulative methane emissions at 100 DAT) were lower in the KH plots than in the CT plots by 23.5% for the 0N plots and 17.2% for the 8N plots (*P*<0.05). Nitrogen fertilization did not significantly affect cumulative methane emissions (Fig. 2b).

**Fig. 3.**
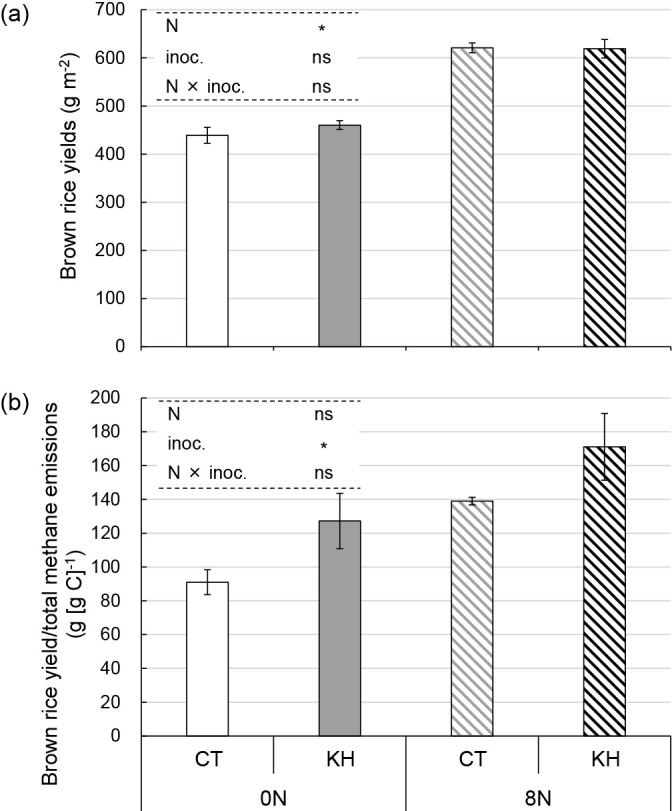
Brown rice yields (a) and brown rice yield/total methane emissions (b). Bars represent standard errors (*n*=3). Asterisks above columns indicate a significant difference for each factor tested using a mixed linear model (*P*<0.05). ns indicates no significant difference. 0N, 0‍ ‍g N m^–2^; 8N, 8‍ ‍g N m^–2^; CT, no inoculation; KH, KH32C inoculation; N, nitrogen fertilization regimen; inoc., KH32C inoculation.

The brown rice yield of the 8N plots was significantly higher than that of the 0N plots (*P*<0.05; Fig. 3a). Rice shoot biomass at 27, 54, 77, and 89 DAT was also significantly higher in the 8N plots than in the 0N plots (*P*<0.05; Fig. S3). No significant differences were observed between the CT and KH plots for brown rice yield (Fig. 3a) or shoot biomass (Fig. S3). Brown rice yield/total methane emissions were significantly higher in the KH plots than in the CT plots (*P*<0.05). Nitrogen fertilization did not exert significant effects (Fig. 3b).

### Transcript copy numbers of *mcrA* and *pmoA* in soil cDNA

**Fig. 4.**
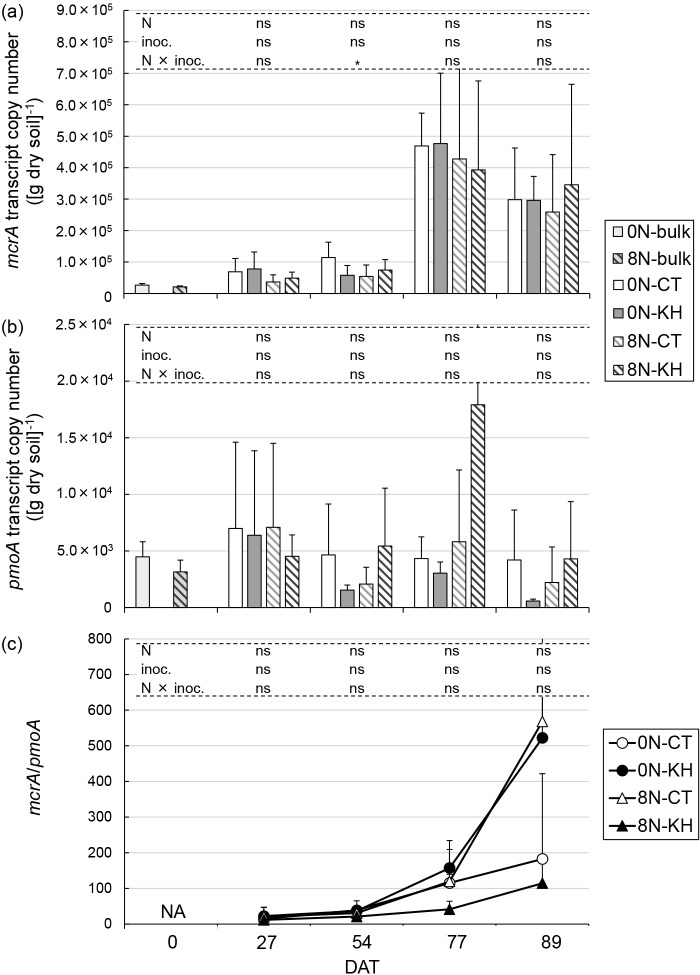
Transcript copy numbers of *mcrA* (a) and *pmoA* (b), and the transcript copy number ratio of *mcrA*/*pmoA* in soil cDNA. Bars represent the standard deviation (*n*=3). Asterisks above columns indicate a significant difference for each factor tested using a mixed linear model (*P*<0.05). ns indicates no significant difference. At 0 DAT (transplantation day), soil samples (bulk) were collected from three random positions in the 0N and 8N plots. At 27 DAT (tillering stage), 54 DAT (panicle initiation stage), 77 DAT (heading stage), and 89 DAT (mid-ripening stage), soil samples were collected from three positions at different distances from a hill. The soil samples from three positions in each plot were pooled and mixed. 0N, 0‍ ‍g N m^–2^; 8N, 8‍ ‍g N m^–2^; CT, no inoculation; KH, KH32C inoculation; N, nitrogen fertilization regimen; inoc., KH32C inoculation; NA, not available.

In quantitative PCR assays, approximately 500-bp amplicons of *mcrA* and *pmoA* were observed using the ME3MF_I/ME2mod and A189/mb661 primer sets, respectively (Fig. S4). The transcript copy number of *mcrA* was the lowest at 0 DAT, and was higher at 27 DAT and 54 DAT than at 0 DAT under all conditions. The transcript copy number of *mcrA* was the highest at 77 DAT across all sampling days, followed by that at 89 DAT. The nitrogen fertilization regimen×KH32C inoculation interaction was significant at 54 DAT (*P*<0.05), but not at 27, 77, or 89 DAT, (Fig. 4a). On the other hand, the transcript copy number of *pmoA* was similar across different sampling days and lower than that of *mcrA*. There were no significant differences on any sampling day (Fig. 4b). The transcript copy number ratio of *mcrA*/*pmoA* increased from 77 DAT. At 89 DAT, the ratios for 0N-KH and 8N-CT increased to more than 500, whereas those for 0N-CT and 8N-KH were less than 200. There was no significant difference in the *mcrA*/*pmoA* ratio on any sampling day (Fig. 4c).

### The composition of *mcrA* and *pmoA* in soil cDNA

**Fig. 5.**
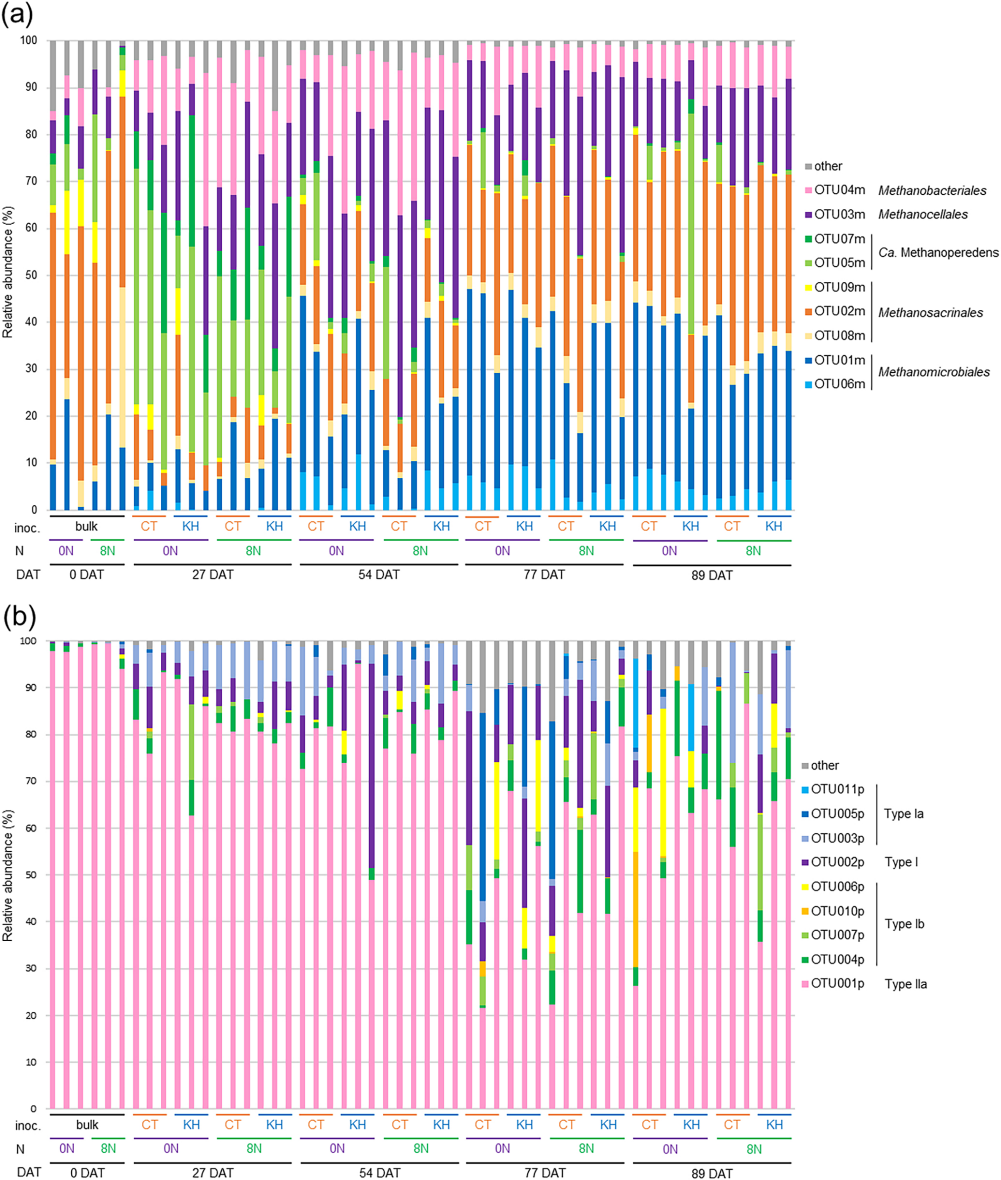
The relative abundance of OTUs of *mcrA* (a) and *pmoA* (b). bulk, no root soil; CT, no inoculation; KH, KH32C inoculation; 0N, 0‍ ‍g N m^–2^; 8N, 8‍ ‍g N m^–2^.

A total of 814,993 high-quality sequences and 91 OTUs and 1,548,044 high-quality sequences and 439 OTUs were obtained in the *mcrA* and *pmoA* amplicon sequences, respectively, of 54 soil cDNA samples. In the case of *mcrA*, 9 major OTUs, OTU01m–OTU09m, occupied 85.0–99.6% in each sample (Fig. 5a). The relative abundance of the 9 major OTUs was mainly affected by the sampling day. OTU04m, identified as a *Methanobacterium* archaeon according to the phylogenetic tree (Fig. S5), was abundant at 27 DAT (5.8–32.8%) and 54 DAT (5.8–31.6%). The *Methanocella* archaeon, OTU03m, was abundant at 54 DAT (16.7–43.0%). The *Ca.* Methanoperedens archaea, OTU07m and OTU05m, occupied 3.1–27.9 and 7.8–50.2% at 27 DAT, respectively. OTU02m and OTU01m increased from 54 DAT, and markedly at 77 and 89 DAT. OTU02m, a *Methanothrix* archaeon in the family *Methanosacrinales*, occupied 26.2–54.1, 19.8–34.6, and 14.4–38.1% at 0, 77, and 89 DAT, respectively. OTU01m, which was in the *Methanoregula* cluster, was abundant at 77 DAT (14.5–40.3%) and 89 DAT (17.1–39.0%) (Fig. 5a and S5). In the case of *pmoA*, 9 major OTUs, OTU001p–OTU007p, OTU010p, and OTU011p, accounted for 82.9–99.9% of the relative abundance of each sample (Fig. 5b). Two phylogenetic groups of methanotrophic bacteria were detected as Type I, including the class *Gammaproteobacteria*, and Type II, including the class *Alphaproteobacteria* (Semrau *et al.*, 2010) (Fig. S6). OTU001p, identified as the genus *Methylocystis* in Type II methanotrophic bacteria, was the most abundant and occupied 94.0–99.4, 62.6–93.4, 49.0–95.1, 21.6–81.6, and 26.3–86.6% at 0, 27, 54, 77, and 89 DAT, respectively. The relative abundance of Type I methanotrophic bacteria was higher at 77 and 89 DAT than at 0, 27, and 54 DAT. *pmoA* sequences in soil cDNA were more diverse at 77 and 89 DAT than at 0, 27, and 54 DAT. The Type I *Methylococcaceae* methanotrophs, OTU005p and OTU002p, constituted 0.2–40.2 and 6.5–28.5% at 77 DAT, respectively. The Type Ib *Methylococcaceae* methanotroph, OTU004p, increased from 0 DAT to 89 DAT, and reached 0.0–23.2% at 89 DAT (Fig. 5b and S6).

**Fig. 6.**
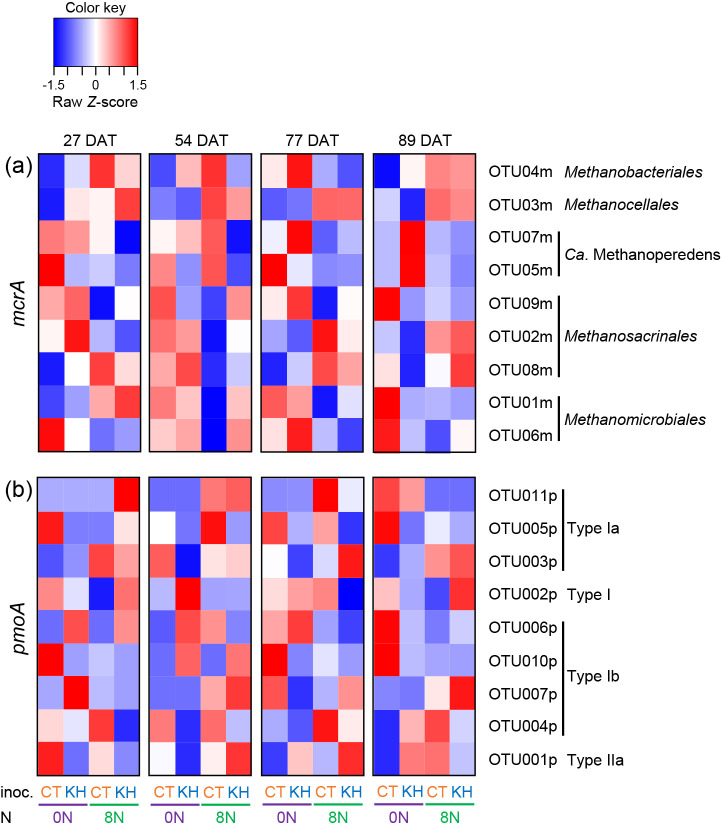
Heatmap based on *Z*-scores of the relative abundance of major OTUs of *mcrA* (a) and *pmoA* (b). *Z*-scores were calculated for each sampling day. CT, no inoculation; KH, KH32C inoculation; 0N, 0‍ ‍g N m^–2^; 8N, 8‍ ‍g N m^–2^.

Heatmap ana­lyses showed that nitrogen fertilization and the KH32C inoculation both affected the relative abundance of the major OTUs of *mcrA* (Fig. 6a). The relative abundance of OTU03m at all investigated stages was higher in the 8N plots than in the 0N plots. In comparisons of the KH plots with the CT plots at all investigated stages, the relative abundance of OTU04m increased for the 0N plots and decreased for the 8N plots. OTU02m was lower at 27 and 54 DAT and higher at 77 and 89 DAT in the 8N plots than in the 0N plots. The relative abundance of OTU02m in the 0N plots at 54, 77, and 89 DAT and in the 8N plots at 77 DAT were lower in the KH plots than in the CT plots. OTU01m was more abundant in the 0N plots than in the 8N plots from 54 DAT to 89 DAT. The relative abundance of OTU01m in the 0N plots from 54 DAT to 89 DAT was lower in the KH plots than in the CT plots. The relative abundance of the *Ca.* Methanoperedens OTUs, OTU07m and OTU05m, was lower in the KH plots than in the CT plots at 27 DAT. In the case of *pmoA* (Fig. 6b), the most abundant *pmoA* OTU, OTU001p did not shown any consistent change associated with the KH32C inoculation or nitrogen fertilization regimen. The relative abundance of OTU005p was lower in the KH plots than in the CT plots in both the 0N and 8N plots at all investigated stages, except in the 8N plots at 27 DAT. In comparisons of the KH plots with the CT plots, the relative abundance of OTU004p decreased at all investigated stages in both the 0N and 8N plots, except at 89 DAT in the 0N plots.

### Correlations between methane flux and other observations

Spearman’s rank correlation coefficient of the relative abundance of the major *mcrA* and *pmoA* OTUs and methane flux showed that some major OTUs were associated with methane emissions. The relative abundance of OTU02m, OTU08m, OTU01m, and OTU06m positively correlated with methane flux, whereas that of OTU04m, OTU03m, OTU07m, and OTU05m negatively correlated with methane flux. The relative abundance of OTU005p, OTU006p, OTU010p, OTU007p, and OTU004p positively correlated with methane flux, whereas that of OTU001p negatively correlated with methane flux (Table 1).

Methane flux, rice shoot biomass, dissolved methane, DOC, Fe (II) concentrations in the soil solution, the copy number of *mcrA*, and the *mcrA*/*pmoA* ratio positively correlated with each other. These factors slightly increased from the tillering stage to the mid-ripening stage (Fig. 1, 2, 4, and S3). The copy number of *pmoA* negatively correlated with the *mcrA*/*pmoA* ratio only (Table 2).

## Discussion

The contribution of rice paddies to global warming is of increasing concern as a major methane emission source. Masuda *et al.* (2018) recently reported the composition of *mcrA* and *pmoA* transcripts in paddy soil, and a compositional ana­lysis of the *mcrA*, *pmoA*, and 16S rRNA genes was performed (Lee *et al.*, 2014; Yuan *et al.*, 2018; Dang *et al.*, 2021). However, there were limitations in the study that revealed the compositional shift of methanogens and methanotrophs in paddy soil (Lee *et al.*, 2014). Additionally, no clear data were presented on the relationships between the soil methanogen and methanotroph communities and methane emissions. The present study using functional gene meta-amplicon sequence and correlation ana­lyses indicated that the compositional shift in the transcripts of *mcrA* and *pmoA* over the waterlogged period was influenced by the KH32C inoculation of rice seeds and provides a more detailed understanding of the microbial regulation of methane emissions in paddy soil. The results obtained will facilitate research on microbial-related methane emissions from rice paddy fields.

The *mcrA*-amplicon sequencing ana­lysis clearly showed that OTU07m and OTU05m of the *Ca.* Methanoperedens archaea, known anaerobic methanotrophic archaea (Haroon *et al.*, 2013), were predominant at 27 DAT (Fig. 5a) and negatively correlated with methane flux (Table 1). Therefore, these OTUs appear to be more closely related to lower methane flux at the tillering stage than that at the booting and mid-ripening stages (Fig. 2a). The relative abundance of OTU03m, belonging to the genus *Methanocella*, increased in nitrogen-fertilized plots (Fig. 6). An increase in the relative abundance of the genus *Methanocella* with the use of chemical nitrogen (urea) fertilizer in rhizosphere soil was also reported by Yuan *et al.* (2018), indicating that *Methanocella* responds to nitrogen fertilizer input. The *pmoA*-amplicon sequencing ana­lysis clearly showed the dominance of OTU001p, belonging to the genus *Methylocystis*, which is widely detected in the paddy field environment, including paddy soil (Lee *et al.*, 2014; Masuda *et al.*, 2018), rhizosphere (Ma *et al.*, 2010; Bao *et al.*, 2014b), and rice roots (Bao *et al.*, 2014a) (Fig. 5b). Henckel *et al.* (2000) demonstrated that the population structure of Type I methanotrophic bacteria changed faster in response to methane concentrations than that of Type II methanotrophic bacteria. The present results indicated that the dissolved methane concentration in the soil solution increased after 72 DAT (Fig. 1a), which may have induced the increase observed in the relative abundance of Type I methanotrophic bacteria from 77 DAT (Fig. 5b). The correlation ana­lysis revealed that the relative abundance of the major OTUs of *mcrA* (OTU01m–OTU08m) and *pmoA* (OTU001, OTU004p–OTU007p, and OTU010p) correlated with methane flux (Table 1). In the case of the KH32C inoculation, the heatmap ana­lysis revealed a decrease in the relative abundance of two *mcrA* OTUs (OTU02m and OTU01m) and two *pmoA* OTUs (OTU005p and OTU004p), which positively correlated with methane flux (Fig. 6), suggesting that the KH32C inoculation recruited a microbial community that suppressed high methane synthesis and low methane oxidization.

According to our broad ana­lysis, a methane emission process appears to be associated with rice growth. Since photosynthates are produced during rhizodeposition (Lu *et al.*, 2000), the increasing rice plant biomass with growth, as shown in Fig. S3, resulted in a gradually elevated DOC concentration in the soil solution (Fig. 1b). These increases in the DOC concentration (Fig. 1b) and soil reduction levels due to continuous waterlogging (Fig. 1c) enhanced methane synthesis, as suggested by the higher transcript copy number of *mcrA* at 77 DAT (heading stage; Fig. 4a), and the higher dissolved methane concentration increased methane flux after 72 DAT (booting stage; Fig. 1a and 2a). The present results are consistent with previous findings (Cheng *et al.* [2006], Lee *et al.* [2014], and Lu *et al.* [2000]). As shown in Fig. 1c, a lower Fe (II) concentration in the soil solution was observed at 100 DAT than at 89 DAT despite the continuous flooding of the paddy field. Tokida *et al.* (2010) conducted *in situ* paddy soil incubation experiments, and indicated that electron-donor consumption (estimated by the quantification of soil organic matter decomposition) due to methane production was more dominant than that by ferric iron (Fe [III]) reduction after the panicle formation stage. The lower activity of Fe (III) reduction to Fe (II) may explain the further increases observed in methane flux and dissolved methane concentrations in the soil solution at 100 DAT. Furthermore, the increase in root decomposition during the ripening stage is generally considered to contribute to methane emissions (Neue *et al.*, 1997; Tokida *et al.*, 2010).

The effects of the KH32C inoculation and nitrogen fertilization on methane emission processes at each rice growth stage investigated were not explained by the suggested process associated with rice growth. Although the KH32C inoculation decreased methane emissions (Fig. 2), the KH32C inoculation did not markedly affect the rice plant biomass (Fig. S3), soil solution chemical properties (Fig. 1), or transcript copy numbers of *mcrA* and *pmoA* in soil cDNA (Fig. 4). Nitrogen fertilization increased the rice plant biomass (Fig. S3), but did not markedly affect soil solution chemical properties (Fig. 1), the transcript copy numbers of *mcrA* and *pmoA* in soil cDNA (Fig. 4), or methane emissions (Fig. 2). The gene copy number ratio of *mcrA*/*pmoA* has been reported to correlate with methane emissions (Yuan *et al.*, 2018). Lee *et al.* (2014) suggested a normalized *mcrA*/*pmoA* transcript ratio (*mcrA* transcripts/*mcrA* genes divided by *pmoA* transcripts/*pmoA* genes) as a parameter for predicting methane flux. The present results showed that the *mcrA*/*pmoA* transcript ratio may explain the increase in methane flux associated with rice growth, but not at the same rice growth stage. In the 8N plots, the lower *mcrA*/*pmoA* ratio in the KH plots than in the CT plots across all sampling days indicated the mitigation of methane emissions by the KH32C inoculation. On the other hand, in the 0N plots, the *mcrA*/*pmoA* ratio was higher in the KH plots than in the CT plots, indicating that the transcript copy numbers of *mcrA* and *pmoA* were not the only factor regulating methane emissions because the KH32C inoculation decreased methane emissions in both fertilization regimens (Fig. 2). Taken together with the results of the amplicon sequencing and correlation ana­lyses, we revealed that the relative abundance‍ ‍of the methanogenic OTUs of *Methanothrix* (an archaeon in the *Methanosacrinales* family), *Methanoregula* (an archaeon within the *Methanomicrobiales* family), and Type Ia and Type Ib *Methylococcaceae* methanotrophic bacteria were responsible for the regulation of methane emissions at the same rice growth stage.

The colonization of bacteria on plants influences plant metabolic pathways and root exudates (Kandaswamy *et al.*, 2019; Liu *et al.*, 2020). Differences in the composition and quantity of root exudates are considered to shape microbial communities and functions in the rhizosphere (Philippot *et al.*, 2013; Liu *et al.*, 2020). We previously demonstrated that KH32C was able to colonize rice seedling roots (Sakoda *et al.*, 2019). The stimulation of rice by KH32C colonization presumably affected metabolic pathways and rice root exudates, thereby recruiting the methane-mitigating microbial community. Therefore, additional mole­cular genetic studies are needed to elucidate the effects of the KH32C inoculation on rice plant-related “priming”.

Collectively, our previous findings and the present results support biogeochemical cycle optimization in agricultural soil through rice plant stimulation by “priming” using the KH32C inoculation of rice seeds. The development of methods for the mitigation of methane emissions from paddy fields is an important step to reduce global warming. The present study showed that the cultivation of rice inoculated with KH32C decreased total rice paddy methane emissions by approximately 20% while maintaining rice grain yield and plant biomass (Fig. 2, 3, and S3). Moreover, Gao *et al.* (2017) reported that KH32C decreased nitrous oxide (N_2_O) emissions, another significant greenhouse gas, from upland soil through its strong N_2_O-reducing ability. The present results will contribute to the development of new applications of the genus *Azoarcus* in the class *Betaproteobacteria* to the mitigation of greenhouse gas emissions from agricultural soil.

## Citation

Sakoda, M., Tokida, T., Sakai, Y., Senoo, K., and Nishizawa, T. (2022) Mitigation of Paddy Field Soil Methane Emissions by Betaproteobacterium *Azoarcus* Inoculation of Rice Seeds. *Microbes Environ ***37**: ME22052.

https://doi.org/10.1264/jsme2.ME22052

## Supplementary Material

Supplementary Material

## Figures and Tables

**Table 1.** Spearman’s correlation coefficient of the relative abundance of major OTUs of *mcrA* and *pmoA* with methane flux.

*mcrA*	
OTU04m	–0.433**
OTU03m	–0.318*
OTU07m	–0.656**
OTU05m	–0.529**
OTU09m	–0.177
OTU02m	0.784**
OTU08m	0.563**
OTU01m	0.537**
OTU06m	0.293*
*pmoA*	
OTU011p	0.107
OTU005p	0.526**
OTU003p	–0.255
OTU002p	0.074
OTU006p	0.341*
OTU010p	0.452**
OTU007p	0.466**
OTU004p	0.398**
OTU001p	–0.620**

The single asterisk and double asterisks indicate a correlation at *P*<0.05 and *P*<0.01 using Holm’s method, respectively (*n*=48).

**Table 2.** Spearman’s rank correlation coefficient of methane flux, shoot biomass, chemical properties of the soil solution, and copy numbers of *mcrA* and *pmoA*.

	Shoot biomass	Dissolved methane	DOC	Fe (II)	*mcrA*	*pmoA*	*mcrA*/*pmoA*
Methane flux	0.783**	0.863**	0.713**	0.770**	0.696**	–0.078	0.669**
Shoot biomass		0.779**	0.803**	0.880**	0.686**	–0.142	0.683**
Dissolved methane			0.845**	0.911**	0.736**	–0.117	0.711**
DOC				0.913**	0.723**	–0.202	0.777**
Fe (II)					0.717**	–0.221	0.776**
*mcrA*						0.275	0.647**
*pmoA*							–0.519**

Double asterisks indicate a correlation at *P*<0.01 using Holm’s method (*n*=48). DOC, dissolved organic carbon.
