# Supplementary material for: Mitigation of Paddy Field Soil Methane Emissions by Betaproteobacterium Azoarcus Inoculation of Rice Seeds
Source: Microbes Environ. 2022 Dec 14;37(4):ME22052. doi: 10.1264/jsme2.ME22052 (PMC9763044; doi:10.1264/jsme2.ME22052)
Supplement: Supplementary file 1 — Supplementary Material [file 37_22052_s1.pdf]

(a)

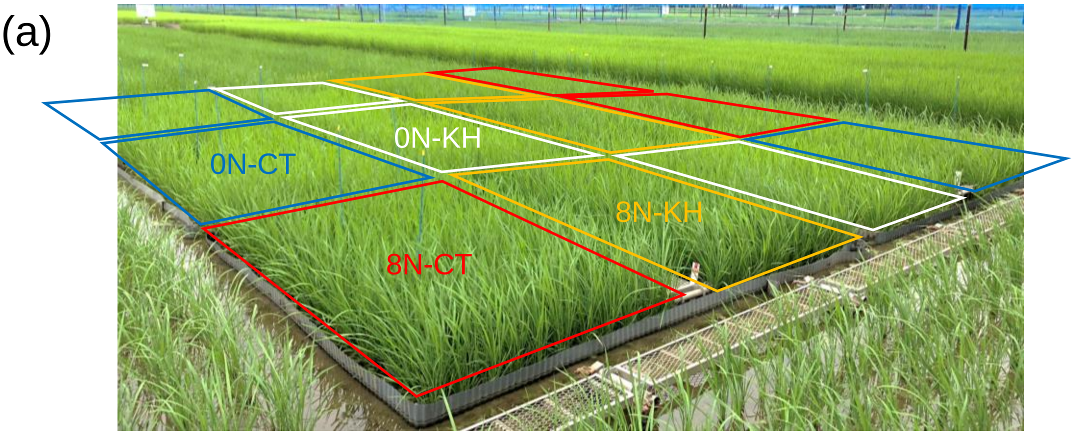

(b)

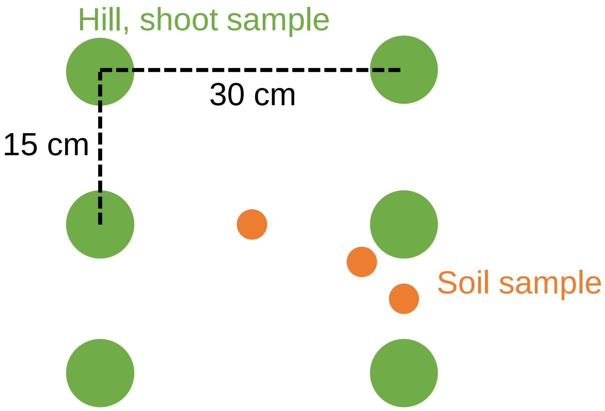

Fig. S1 Rice cultivation plot (a) and the positions of soil sampling (b).

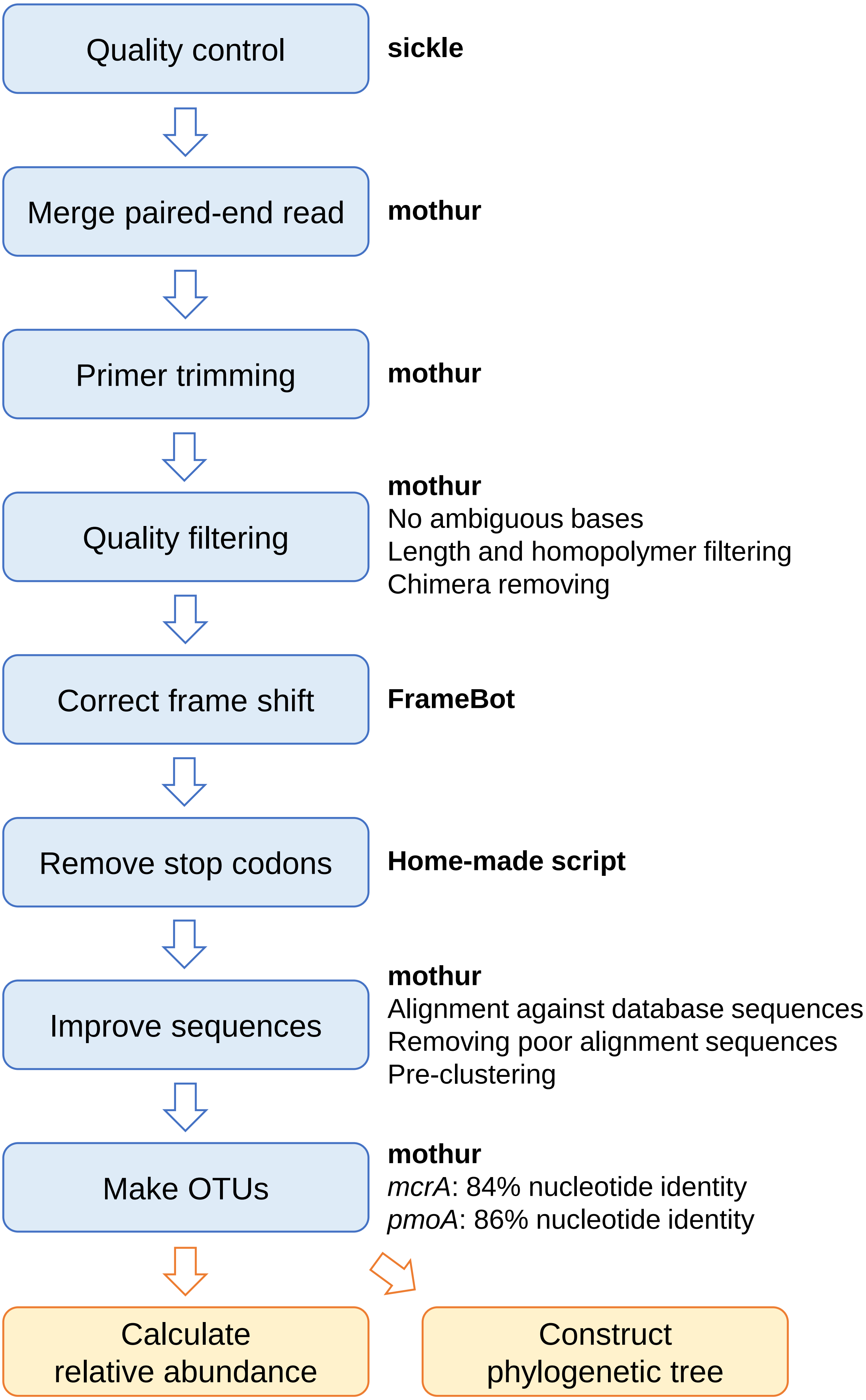

Fig. S2 The procedure of sequence data analysis.

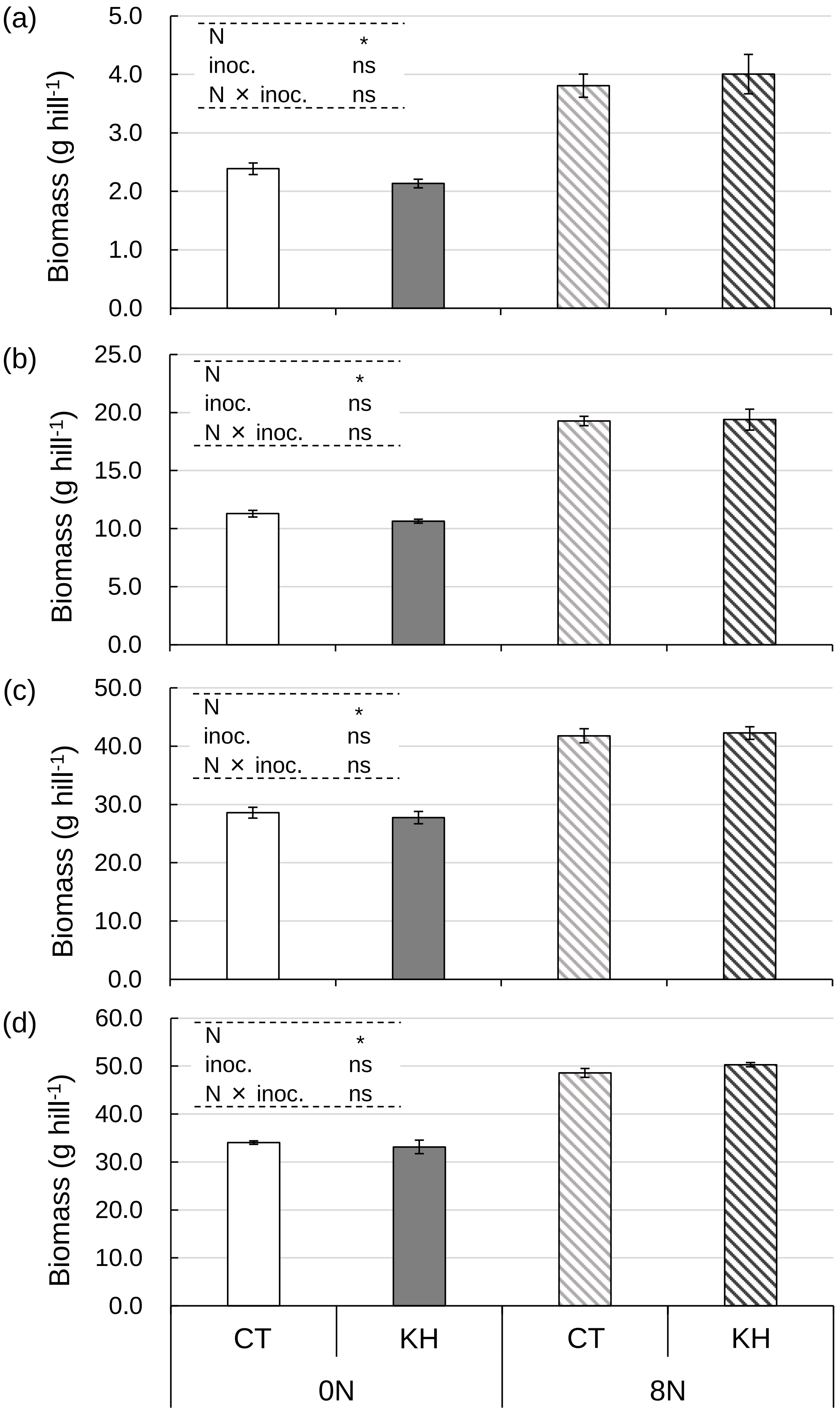

Fig. S3 Rice plant shoot biomass at 27 DAT (tillering stage; a), 54 DAT (panicle initiation stage; b), 77 DAT (heading stage; c), and 89 DAT (mid-ripening stage; d). The bars represent standard errors (n=3). Asterisks above the columns indicate a significant difference for each factor tested using a mixed linear model (P<0.05). ns indicates no significant difference. 0N, 0 g N m^{-2}; 8N, 8 g N m^{-2}; CT, no inoculation; KH, KH32C inoculation; N, nitrogen fertilization regimen; inoc., KH32C inoculation.

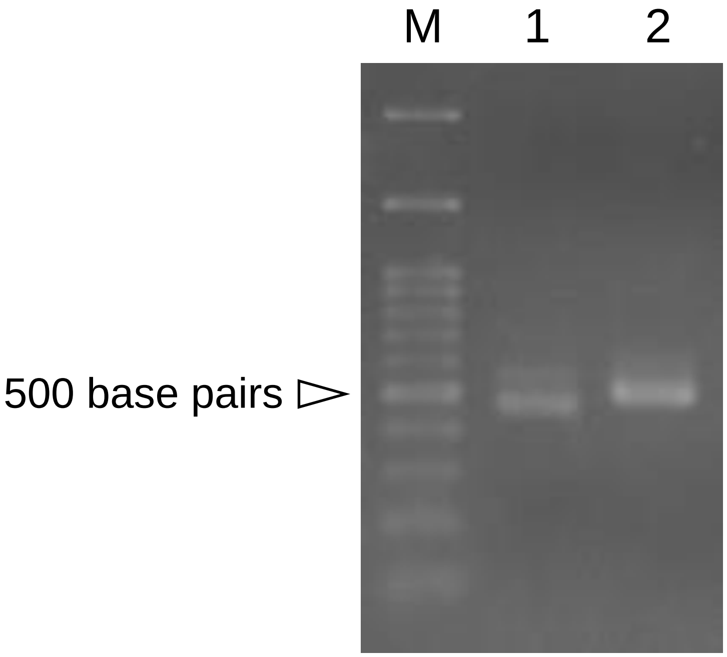

Fig. S4 Agarose gel electrophoretic patterns of PCR amplification of soil cDNA using primer sets of ME3MF_I/ME2mod and A189/mb661. 1, ME3MF_I/ME2mod; 2, A189/mb661; M, DNA marker.

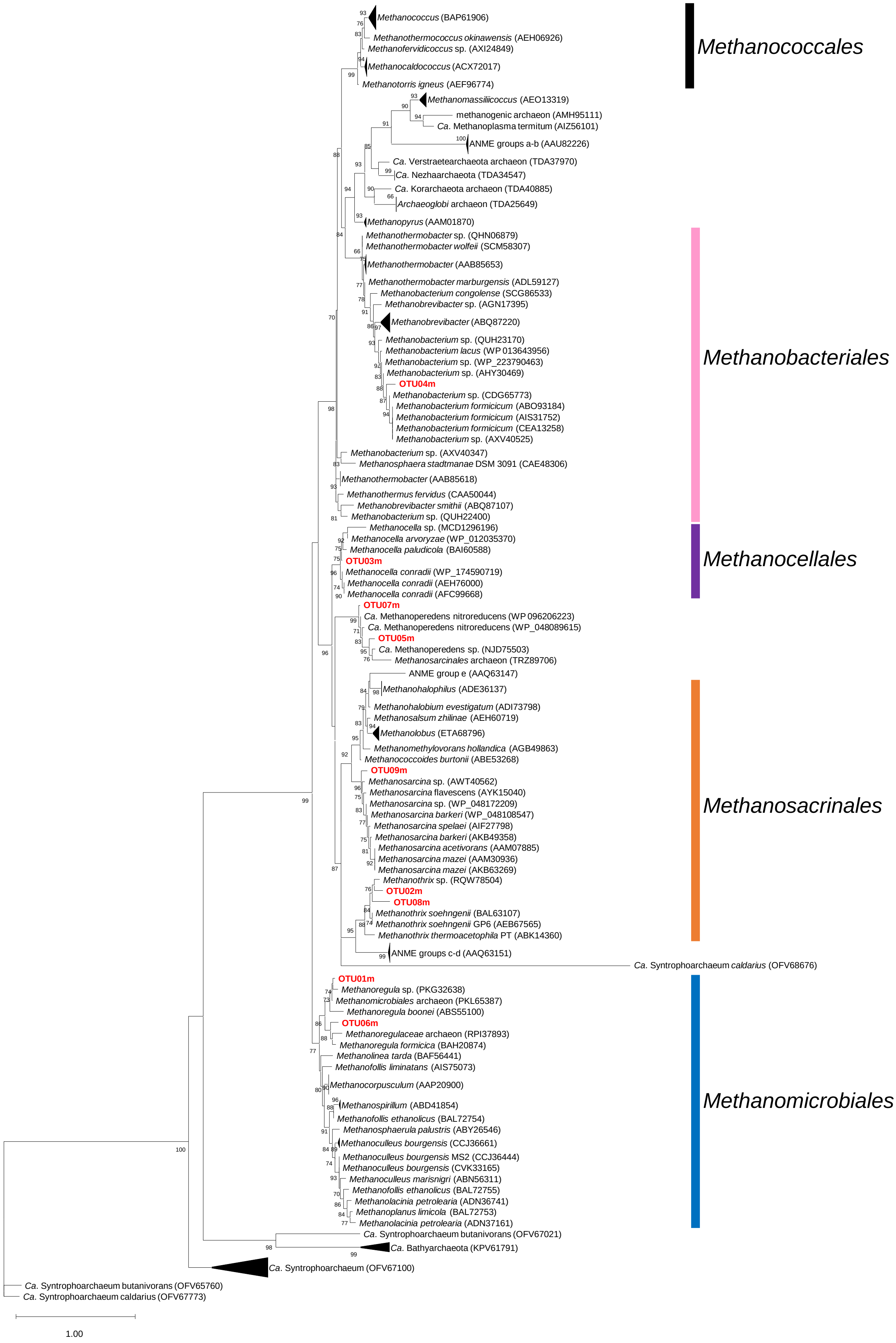

Fig. S5 Maximum-likelihood tree of *mcrA* amino acid sequences. The red-colored OTU sequences were obtained in this study. The tree was built based on 128 amino acid positions using a MtZoa amino acid substitution model. Node numbers indicate SH-like aLRT ≥70.

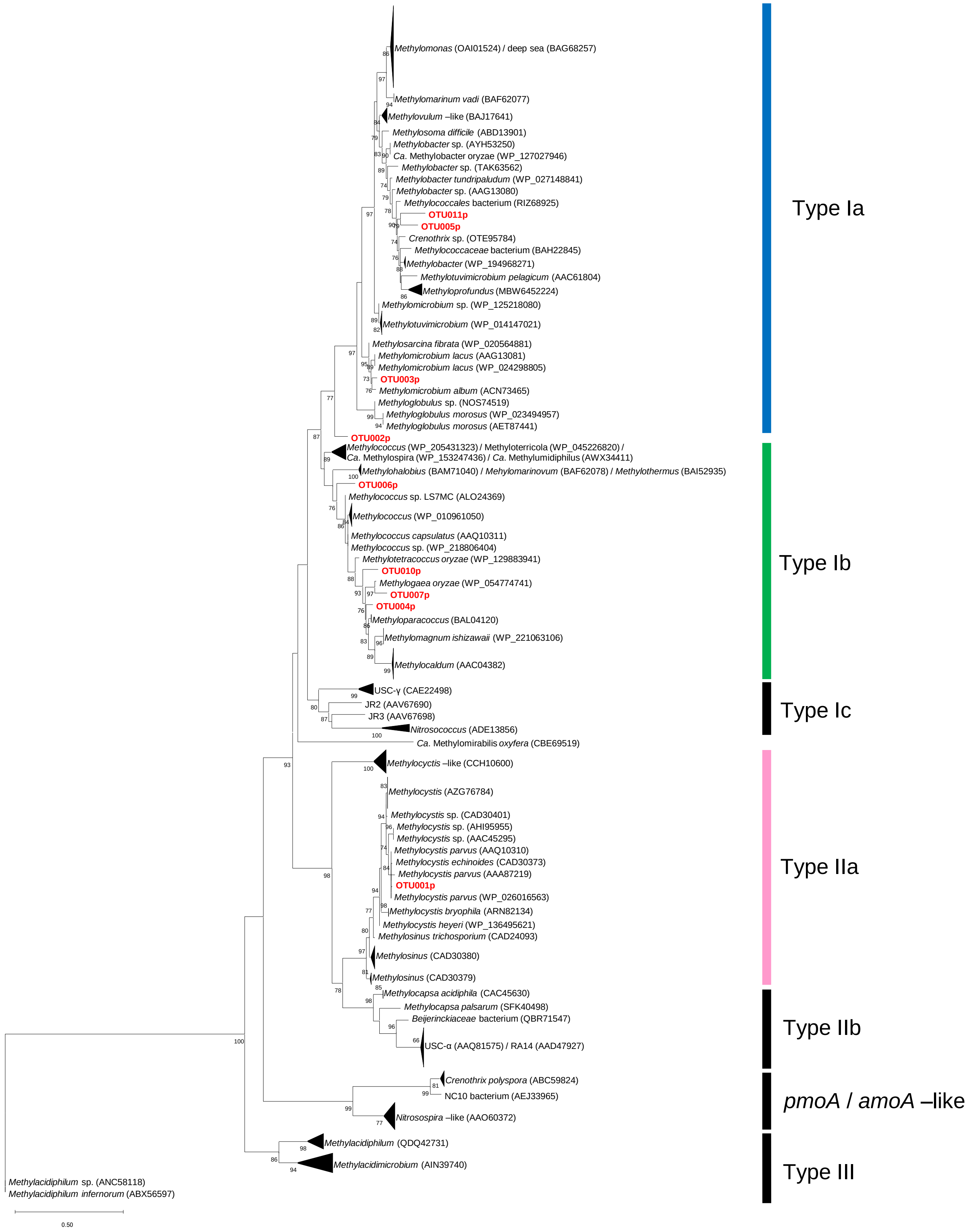

Fig. S6 Maximum-likelihood tree of *pmoA* amino acid sequences. The red-colored OTU sequences were obtained in this study. The tree was built based on 157 amino acid positions using an LG amino acid substitution model. Node numbers indicate SH-like aLRT ≥70.
